# Architects and Sanatoria

**Published:** 1905-07-08

**Authors:** E. T. Hall


### Editor's Letter-Box.

ARCHITECTS AND SANATORIA.

MR. E. T. HALL, F.R.I.B.A., writes: I have read with interest the article in your last issue on "Architects and Sanatoria." In the first place, on behalf of my profession, I must take exception to the suggestion that the cost of sanatoria is influenced by the question of the architect's fees. Among the architects whose acquaintance I have the honour of possessing—and I know a very large number—I am perfectly certain that their own remuneration does not enter, even in the remotest degree, into the question of their duty to their clients. If an architect could design a building containing everything he is required to provide, at half the cost of a similar building designed by another architect, the first knows quite well that he would be likely to get ten times the work of the second, and therefore on "a business basis" his interest is to make the building as cheap as he can, consistent with his other duties to his client.

Many speakers at the Sanitary Institute referred to temporary châlets which had cost £80, £40, £35, and less per patient. I have no doubt these are all perfectly accurate figures.

There is at my Frimley Sanatorium a shelter large enough to take a patient but used as a workshop, which cost 7s. 6d., the labour being gratuitous. It is made of unhewn posts, with a waterproof roof, the sides being of interlaced boughs—a primitive shelter such as our remote ancestors built. At the other end of the chain is a sanatorium which, if rumour speaks aright, is costing something like £1,000 a bed. Between these two extremes there are links of varying financial dimensions.

We must, in considering this question, bear in mind that there is as great a difference between a sanatorium of temporary and one of permanent buildings, as between a military camp of huts and a depot of permanent buildings like the new one on Salisbury Plain. The two are not comparable.

The question of fire, too, is one not to be ignored when you are building among pinewoods, because inflammable buildings once alight would be more than likely to set fire to the whole district. There have been two or three forest fires in the Frimley district. It is almost impossible to put them out in dry weather, and they burn until the fire comes to a clearing.

Again, the cost of the patients' building is relatively a small portion of the whole cost of an institution. The administration buildings, including the laundry with its machinery, engine-houses, electric generating plant, boilers, steam- and hot-water pipes, roads and paths, drains and sewer, water supply, etc., are the items which bring up the capital cost. The engineering work alone of a large sanatorium or hospital costs £60 to £80 a bed, but such an outlay reduces the annual maintenance charges to a far greater extent than the normal interest on the outlay.

At Heatherside the roads, paths, drains, and sewer (about two miles long) cost nearly £45 a bed.

Another element in outlay is the number of beds per room. In a permanent building, if ten patients are put in one room it is manifest that with an equal cubic space per patient, that room will cost infinitely less than if only one person were put in a room, in the same way that an empty box or cupboard costs less than one filled with pigeon-holes. Added to this there are the æsthetic considerations. These are held by most cultured people to be of importance, both to the patient and to the public. The effect on a patient of a cheerful, bright, and artistic building, however simple, has its value in the cure and at least as great a value to the public generally. That the design should be simple and inexpensive goes without saying—but art should not be absent.

Again, "necessary accommodation" is a relative expression. I know of a workhouse building, erected about a century ago, which consisted of a series of single rooms of small dimensions—I think as a fact seven feet high—each having a door opening into a quadrangle, neither plastering nor any other covering was on the walls or ceilings, nor were the rooms heated or artificially lighted. The cost of the institution was small but no one at the present day would dream of lodging the poor in such a place, and what applies to a workhouse applies with much greater force to hospitals and sanatoria. If architects are so instructed, they can design a building as you say to cost "£100 per bed, apart from the administration block," but it must then be understood that £100 worth of work can only be obtained.

In conclusion, I am quite sure that architects are only too anxious to assist the public in getting the best results for the smallest practicable outlay. They only need to be instructed as to the standard to which they are to conform.

.·. If Mr. Hall reads our article again he will see that he has misunderstood our suggestions and his limited interpretation does not apply to them. Then, too, the course pursued by Mr. Hall in enumerating many things and plans to be avoided is not helpful in the direction of our search for a wise solution of the present costly and, as we believe, prohibitive plans of sanatoria. We have heard much of the simpler life lately, and we sadly need simplicity in methods of design and construction in the present extravagant days.—ED. THE HOSPITAL.

